# Mantle flow distribution beneath the California margin

**DOI:** 10.1038/s41467-020-18260-8

**Published:** 2020-09-08

**Authors:** Sylvain Barbot

**Affiliations:** Department of Earth Sciences, University of Southern California, Los Angeles, CA 90089-0740 USA

**Keywords:** Geodynamics, Geophysics, Tectonics

## Abstract

Although the surface deformation of tectonic plate boundaries is well determined by geological and geodetic measurements, the pattern of flow below the lithosphere remains poorly constrained. We use the crustal velocity field of the Plate Boundary Observatory to illuminate the distribution of horizontal flow beneath the California margin. At lower-crustal and upper-mantle depths, the boundary between the Pacific and North American plates is off-centered from the San Andreas fault, concentrated in a region that encompasses the trace of nearby active faults. A major step is associated with return flow below the Eastern California Shear Zone, leading to the extrusion of the Mojave block and a re-distribution of fault activity since the Pleistocene. Major earthquakes in California have occurred above the regions of current plastic strain accumulation. Deformation is mechanically coupled from the crust to the asthenosphere, with mantle flow overlaid by a kinematically consistent network of faults in the brittle crust.

## Abstract (web summary)

Although the surface deformation of tectonic plate boundaries is well determined by geological and geodetic measurements, the pattern of flow below the lithosphere remains poorly constrained. Here, the author finds that major earthquakes in California have occurred above the regions of current plastic strain accumulation in the mantle.

## Introduction

Plate tectonics provide a paradigm to understand the shape and dynamics of Earth’s surface. Most plate boundaries involve either divergence, like at oceanic spreading centers and continental rifts, or convergence, such as subduction and collision zones, but a small fraction involves transform boundaries that facilitate plate kinematics on the global sphere. These boundaries accommodate plate-parallel relative plate displacement by strike-slip motion on vertical or steeply dipping faults. The San Andreas fault system in California consists of major strike-slip faults and subsidiary thrusts faults that transfer extension at the East Pacific Rise in the Gulf of California spreading center to the south and the relative motion between the Gorda and Pacific plate along the Mendocino fracture zone to the north. Contemporary surface deformation at the California margin is captured by a dense continuous geodetic observatory^1,2^ and the long-term slip accumulation on numerous faults is well established^3^, providing a unique opportunity to study the mechanics of strain accumulation at a continental transform. Seismological observations, in particular shear wave splitting^4,5^ and seismic anisotropy^6^, provide unique constraints on the orientation of mantle flow, but a cohesive picture of flow distribution below the margin consistent with surface observations is still missing.

The relative motion between plates is accommodated at intra-continental transforms by various mechanisms of deformation. In the brittle layer, fault slip is the dominant mode of strain accumulation and release, mediated by the complex evolution of frictional resistance that leads to stick-slip and the earthquake and slow-slip phenomena^7,8^. The bottom of the seismogenic layer is thought to represent an isotherm for the frictional stability of quartz-rich fault gouge^9^. In California, the maximum depth of seismicity varies from 10 to 20 km depending on location^10^, inversely correlated with the surface heat flow^11^. Faults are steadily creeping below a locking depth that varies depending on the fault segment^12–15^ and often matches the bottom depth of seismicity^16^. Variations of locking depth across the fault system may be attributed to differences in fluid content, geothermal gradients, and stability of fault slip in the seismogenic zone, and may be non-stationary^17^. Immediately below the seismogenic layer, deformation occurs in localized shear zones that creep steadily down to the brittle-ductile transition, below which the deformation becomes plastic and more distributed, controlled by diffusion creep, grain-boundary sliding, and dislocation creep^18,19^.

The depth of the brittle-ductile transition depends on the geothermal gradient, grain-size, frictional strength, and lithology, and is poorly known. Constraints from mineral physics and numerical modeling indicate the presence of a kilometers-wide shear zone below major strike-slip faults^20–24^, as they provide fluid and heat pathways and stress concentrations, all major weakening constituents. Transient deformation following large California earthquakes indicates distributed lower-crustal flow below fault roots, for example, after the 1992 Landers, 1999 Hector Mine, 2004 Parkfield, and 2010 El Mayor-Cucapah earthquakes^25–29^. Geophysical data also provide strong evidence for the mobility of the asthenosphere below the California margin, including far-reaching postseismic deformation^27,30^, the development of seismic anisotropy in the upper mantle^31^, and strong lateral variations of seismic velocity in the lower-crust and upper-mantle below major strike-slip faults in southern California^32^.

The rate of seismicity is controlled by strain accumulation, which is now assimilated to help characterize seismic hazards in California^33,34^. The long-term kinematics of the California margin is constrained by dating of offset markers along plate boundary faults and the relative motion of tectonic plates at geological time scales. Contemporaneous slip-rates are established with geodetic data using a network of buried faults^1,35^ or block modeling^36–40^. These methods are of great practical interest because estimation of fault slip-rate is a direct model outcome^41^. However, they make a number of simplifying assumptions that conflict with our understanding of lithosphere mechanics. For example, block modeling gives rise to unrealistic fault-perpendicular offsets due to block rotation, and fault-based models only consider brittle deformation. These models also have difficulty resolving off-fault deformation, which may account for ~50% of the cumulative strain in the Eastern California Shear Zone^42^. Several alternative approaches relax these assumptions, including deformation of a thin plate with variations of elastic thickness^43,44^ and viscoelastic modeling^45,46^, where viscoelastic flow is limited to the postseismic response of large earthquakes. None of these studies reveal how plastic strain is accumulated in the ductile substrate on an on-going basis.

We seek to image the deep deformation that accrues along the California margin compatible with our understanding of lithosphere rheology. We present a method to image state-wide horizontal mantle flow from geodetic data with minimal assumptions and tradeoffs. At long wavelengths, the tectonic contribution of the velocity field at Earth’s surface during the interseismic period is caused by fault creep in the brittle layer and distributed flow in the viscoelastic substrate. In a viscoelastic medium, any parcel that deforms plastically entrains the surrounding rocks by elastic coupling^47–50^. We exploit this effect to reconstruct the subsurface strain-rate distribution by inversion of the surface velocity field (see Methods section). We find that the San Andreas fault system represents the extension of a 50-km-wide trans-lithospheric shear zone in the brittle crust. Major earthquakes have occurred in regions with rapid plastic strain accumulation and the orientation of the principal strain axes is compatible with the earthquake source mechanisms. The numerous parallel dextral faults in Southern California are associated with a return flow below the Mojave section of the Eastern California Shear Zone.

## Results

### Modeling assumptions

**Fig. 1:**
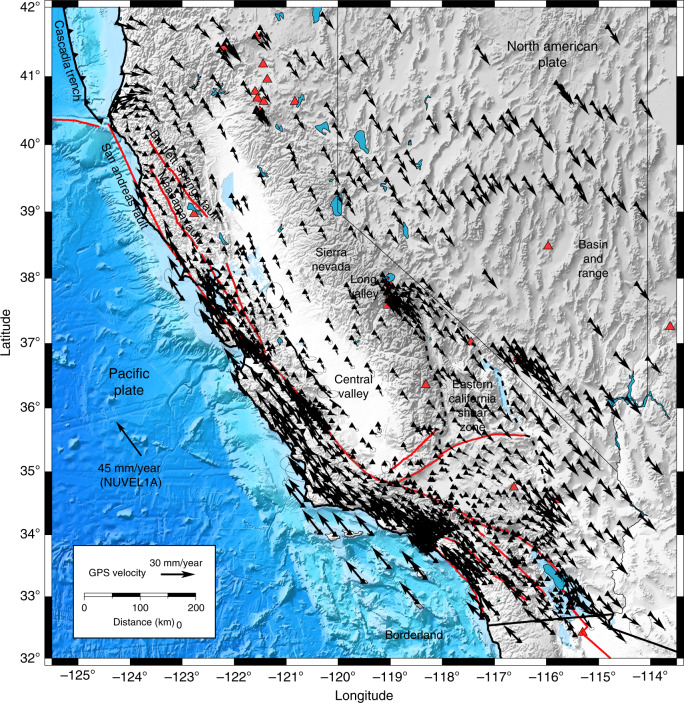
Distribution of the GPS velocity around the San Andreas fault. The GPS velocity field is available at ftp://data-out.unavco.org/pub/products/velocity/. The red triangles correspond to quaternary volcanos. The trace of major faults is from the USGS catalog of quaternary faults https://www.usgs.gov/natural-hazards/earthquake-hazards/faults. The NUVEL1A velocity is for the Pacific plate relative to the North American plate^76^. The dashed contour indicates the approximate extent of the Eastern California Shear Zone.

We obtain the velocity field from the Plate Boundary Observatory through the UNAVCO (unavco.org) repository (Fig. 1). The velocity field is representative of the deformation that accrues for at least 2.5 years up to 15 September 2018, and may include a contribution from the continuing postseismic relaxation of the 1992 Landers, 1999 Hector Mine, and 2010 El Mayor-Cucapah earthquakes. We assume that the deformation is mainly caused by distributed plastic flow in the lower crust and upper mantle. We devise a mesh of 20 × 20 km semi-infinite volume elements that extend down from 20 km depth along a coordinate system aligned with the plate boundary axis, with *x*_1_ and *x*_2_ the parallel and perpendicular axes, respectively. We assume that the viscous flow is horizontal and incompressible. This implies that the vertical plastic strain-rate tensor components are zero and that ${\dot{\epsilon }}_{11}+{\dot{\epsilon }}_{22}=0$. The model ignores vertical shear, which accommodates the absolute motion of the plate boundary in a plume-fixed reference system, as this displacement is undistinguishable from a rigid-body translation at the regional scale. The maximum shear strain-rate is presumably aligned with the plate boundary axis, but small rotations are allowed by considering both ${\dot{\epsilon }}_{12}$ and ${\dot{\epsilon }}_{11}$ free parameters, strictly enforcing ${\dot{\epsilon }}_{22}=-{\dot{\epsilon }}_{11}$ and ${\dot{\epsilon }}_{12}\le 0$ to ensure overall right-lateral shear parallel to the plate boundary and left-lateral shear perpendicular to the plate boundary, and incompressibility. The mesh extends from Baja California, Mexico to north of the Mendocino triple junction, encompassing 74 × 33 = 2442 volume elements.

To enforce kinematic continuity from the locking depth in the crust to the asthenosphere, we allow strike slip at the deep extension of major faults in the brittle layer, from 15 to 20 km. These embedded surface elements include the entire length of the Garlock, San Jacinto, Elsinore faults, a large section of the San Andreas fault from Gilroy in the Bay Area down to the Salton trough, and the southern section of the Calaveras fault. In addition, we allow shallow fault slip in the creeping section of the San Andreas fault, north of Parkfield. Variation of slip velocity on these deep segments reflect changes in locking depth along a fault and the possibility of a gradual transition from locked to creeping, and do not always represent the long-term fault slip-rate. Large deformation occurs around the Long Valley caldera due to volcanic unrest^51^. Instead of removing the surrounding GPS stations, we model this deformation explicitly with shallow volume elements representing shear and volume change associated with a network of dyke, magma chambers, and connected faults.

### Inversion method and resolution

Imaging of localized and distributed deformation can be cast as an inverse problem (see Methods section) where plastic strain-rate in volume elements and slip velocity on surface elements are related to surface GPS velocity vectors by Green’s functions^52–56^. The formulation enforces that all the strain tensor components derive from a single-valued velocity field with conservation of angular and linear momentum in a half space^49,50^. We also simultaneously fit for a rotation and a translation such that the results are independent of the reference frame of the velocity field. The inversion is under-determined with three components for each of the 937 GPS stations, summing up to 2811 data points and 5578 model parameters, among which 588 are dedicated to resolving deformation below Long Valley caldera. Stable inversion results can be obtained with first-order Tikhonov regularization with or without positivity constraints, but all results shown derive from a non-linear inversion that imposes a negative shear strain-rate component ${\dot{\epsilon }}_{12}$.

**Fig. 2:**
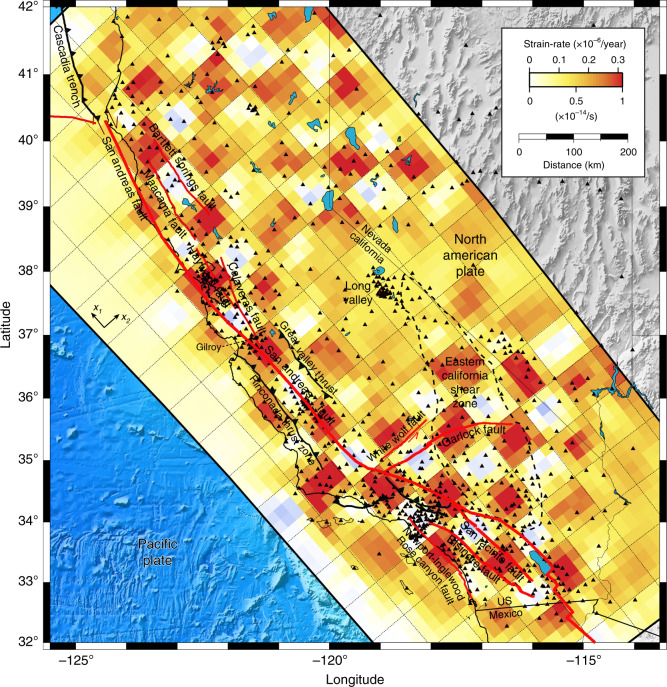
Checkerboard test for inversion of plastic strain-rate. The checkerboard pattern of strain-rate consisting of clusters of four by four neighboring volume elements with non-zero shear strain-rate is indicated by the dashed lines. The recovered pattern after inversion of the synthetic data (black triangles) is in the colored background. Loss of resolution occurs offshore and around the Long Valley Caldera due partly to insufficient station coverage.

The imaging power provided by the Plate Boundary Observatory is shown in a checkerboard test in Fig. 2, indicating that regions of strain accumulation in the lower crust and upper mantle roughly 50 km wide can be resolved appropriately. Loss of resolution in the Sierra Nevada centered on the Long Valley caldera is due to the sparsity of the Plate Boundary Observatory geodetic network in the region and the challenging tradeoffs associated with resolving volcanic unrest and deep mantle flow. Inspection of the resolution matrix reveals a spatial pattern similar to the checkerboard test. As the data offer limited resolution at depth, we do not image how the flow changes across the Moho or how the shear zone may broaden in the asthenosphere. The model therefore represents an average strain accumulation, perhaps most representative of the asthenosphere due to its greater depths and dimensions leaving a broad footprint in the velocity field.

### Flow distribution

**Fig. 3:**
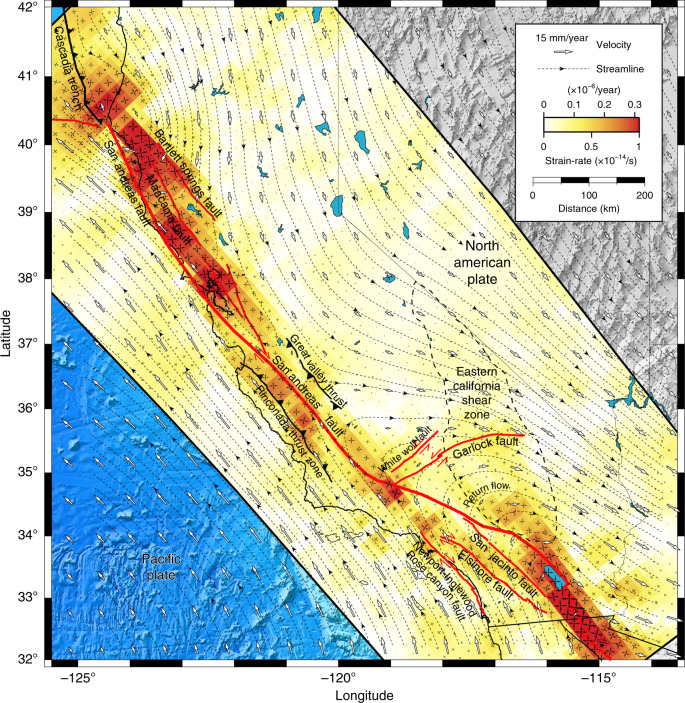
Contribution of viscoelastic flow on plate boundary deformation. The background color indicates the spatial distribution of strain accumulation (the norm of the strain-rate tensor) along the plate boundary. The pairs of black arrows indicate the principal axes of the strain-rate tensor. The white arrows indicate the surface velocity predicted by the model. The dashed black lines with arrows are the flow streamlines. The thick red lines indicate the surface trace of major strike-slip faults. The thick dashed contour indicates the approximative extent of the Eastern California Shear Zone.

The inversion of the Plate Boundary Observatory velocity field reveals the pattern of horizontal flow in the lower crust and upper mantle (Fig. 3). Plastic strain accumulation during the interseismic period is heterogeneous, with large strain-rates of the order of 1 × 10^−14^/s localized below major faults, but considerably smaller far from the plate-boundary faults. Deep shear zones are concentrated underneath major faults, spreading across a 50-km-wide shear zone at the minimum, implying substantial variations of ambient stress or rheological properties across the margin. Strain may be even more localized than currently resolved, but filtering by the elastic crust eventually limits the recovery of finer details. We surmise that the shear zone is narrower in the lower crust, broadening to a wider region in the asthenosphere, following theoretical predictions^20,21^. The deep regions of strain concentration may reflect strain softening due to grain-size evolution, the development of plastic anisotropy, or thermo-mechanical feedbacks with the overlaying brittle section.

Mantle flow is more localized throughout northern and central California, down to the Garlock fault, and in the Salton trough, from the San Bernardino mountains to the East Pacific Rise, with a 120-km-wide restraining step-over separating these two regions. The intervening space corresponds to a broad, spatially coherent shear zone, widely distributed from west to east from the Channel Islands offshore to the Eastern California Shear Zone in the Mojave block, extending as far north as the White Wolf fault and the Little Lake fault zone, across the Garlock fault. Across the central section of the San Andreas fault, the deformation resembles Couette flow on a horizontal plane.

The northern termination of the California margin exhibits a strong horizontal return flow around the Gorda slab, which may overprint an unresolved vertical corner flow due to plunging of the slab below the North American plate. Another return flow in southern California is more widely distributed due to the large extent of the restraining bend in the plate-parallel and plate-perpendicular directions. Flow is more uniform away from the San Andreas fault system. Smaller patches of strain accumulation can be found along Owens Valley, Panamint Valley, and Death Valley, all the way to the Walker Lane in Nevada, but low resolution around Long Valley caldera affects the imaging result in this region. A patchy, low-amplitude, background plastic strain-rate persists throughout the California margin from the Pacific plate to the Basin and Range, presumably reflecting the ambient tectonic stress or small-scale mantle convection driven by density anomalies that are not isostatically compensated^57^.

Throughout California, the plate boundary at depth is not exactly aligned with the surface trace of the San Andreas fault, except in the Central Valley, as previously established from the distribution of surficial geodetic strain-rate^14^. To the north, the shear zone is firmly east of the San Andreas fault, spreading across the Maacama and Bartlett Springs fault systems in Mendocino County and centered on the Hayward, Calaveras, and Greenville fault system underneath the Bay Area. In the Salton trough, a zone of high strain concentration spreads across the Coachella segment of the San Andreas fault and the San Jacinto fault, contiguous to a broad secondary zone of more distributed strain accumulation.

### Residual velocity

The modeling assumptions are compatible with observations at the California margin, with a sum of squares of residuals of (0.3 mm/yr)^2^ on average for each station and an overall variance reduction of 95.5%. The vertical component of velocity is typically smaller and has less influence on the result (Supplementary Fig. 1).

**Fig. 4:**
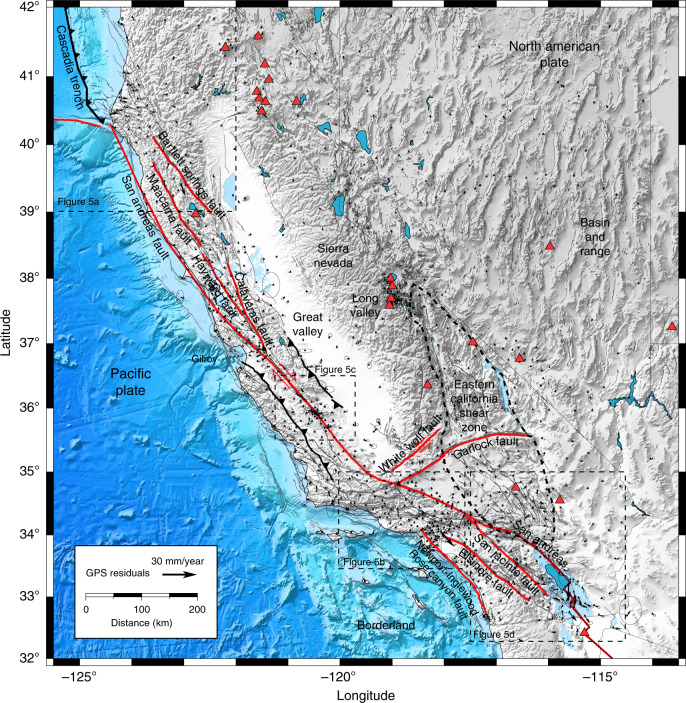
GPS residuals velocity. Residuals are plotted at the same scale as in Fig. 2. The largest spatially coherent residuals occur off the Cascadia trench, around Parkfield, in the Transverse Range, and around the recent 1999 Hector Mine and 2010 El Mayor-Cucapah earthquakes, shown in more detail in Fig. 5. The red triangles correspond to quaternary volcanos.

**Fig. 5:**
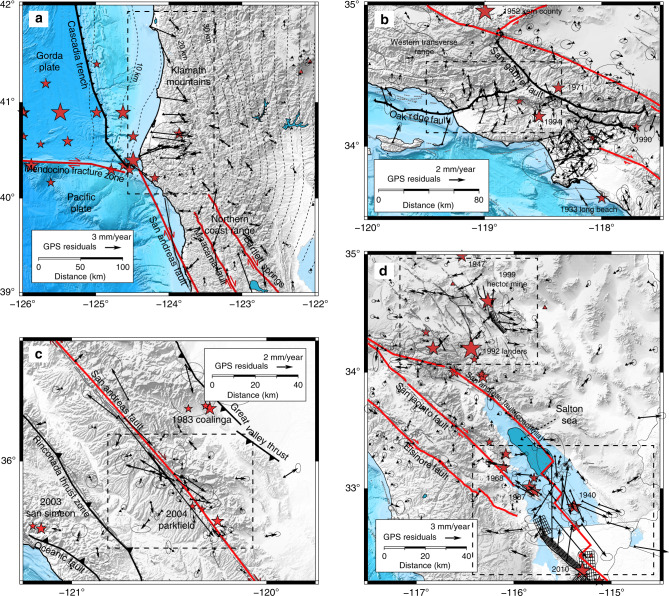
Spatially coherent residuals. **a** Residual velocity in the northern California coast. Off the Gorda plate, the residuals are due to a shallow locked section of the Cascadia trench. The dashed lines are the 5-km contours of the Cascadia megathrust (https://earthquake.usgs.gov/data/slab/models.php). Diverging velocity in the northern Coast Ranges may be due to variation of water storage in aquifers. **b** Residual velocity around the Transverse Range due to shortening occurs across the Oak Ridge fault and the San Gabriel fault and in the Los Angeles Basin due to oil and gas extraction. The black lines with chevrons indicate major thrust faults. **c** Velocity residuals near the Parkfield segment of the San Andreas fault due to complex shallow fault geometry, shallow creep, and afterslip from the 2004 Mw = 6.0 earthquake. **d** GPS residual velocity around the 1999 Mw = 7.1 Hector Mine earthquake and the 2010 Mw = 7.2 El Mayor-Cucapah earthquake in the Salton trough. The red stars indicate the hypocenter of historical earthquakes of magnitude <6 since 1906.

The residuals with the Plate Boundary Observatory velocity field in the horizontal direction are shown in Fig. 4. Systematic and spatially coherent residuals in the horizontal components can be found near the Mendocino triple junction, near the Parkfield segment of the San Andreas fault, in the Transverse Range and the Los Angeles basin, and in the near-field of recent large earthquakes in Southern California, shown in more details in Fig. 5. Strong subsidence in the Central Valley associated with hydrological processes also produce outliers (Supplementary Fig. 1). These residuals occur because of shallow processes not accounted for in the model that cannot be explained by horizontal plastic flow in the lower crust and mantle.

In northern California, the tectonic regime changes from strike-slip faulting on the San Andreas fault system to subduction of the Gorda plate underneath north America at the Cascadia trench. The strain associated with the Gorda down-going slab creates trench-perpendicular residuals around the Klamath mountains in Humboldt County. Other residuals in the Northern Coast Range may be due to hydrological activity^58^. Misfits near Parkfield correspond to small scale variations of creep between the Cholame and creeping segments modulated by postseismic relaxation of the 2004 Parkfield earthquake^59,60^. The shortening centered on the Oak Ridge fault system and the San Gabriel fault in the Los Angeles Basin is due to slip partitioning around the big bend of the San Andreas fault^61,62^ involving shallow thrusts splaying off a deeper sub-horizontal décollement^63^.

Deformation in the Transverse Range therefore represents an example of thin tectonics, i.e., faults confined to the crust without mantle roots, instead of deformation driven by mantle down-welling^57^. Other large residuals in the Los Angeles Basin are due to extraction and injection of groundwater and oil^64^. Finally, the forward model leaves out residuals around recent large earthquakes in southern California due to afterslip and other postseismic processes that were still ongoing in 2018. The postseismic deformation of large strike-slip earthquakes includes vertical shear and short-wavelength features that are not captured in the model. These are most evident in the near-field of the 1999 Hector Mine earthquake and in the Salton trough, north of the El Mayor-Cucapah earthquake rupture, where slip on many shallow faults in the Imperial Valley was accelerated after the mainshock^29^.

### Relation with faulting and seismicity

**Fig. 6:**
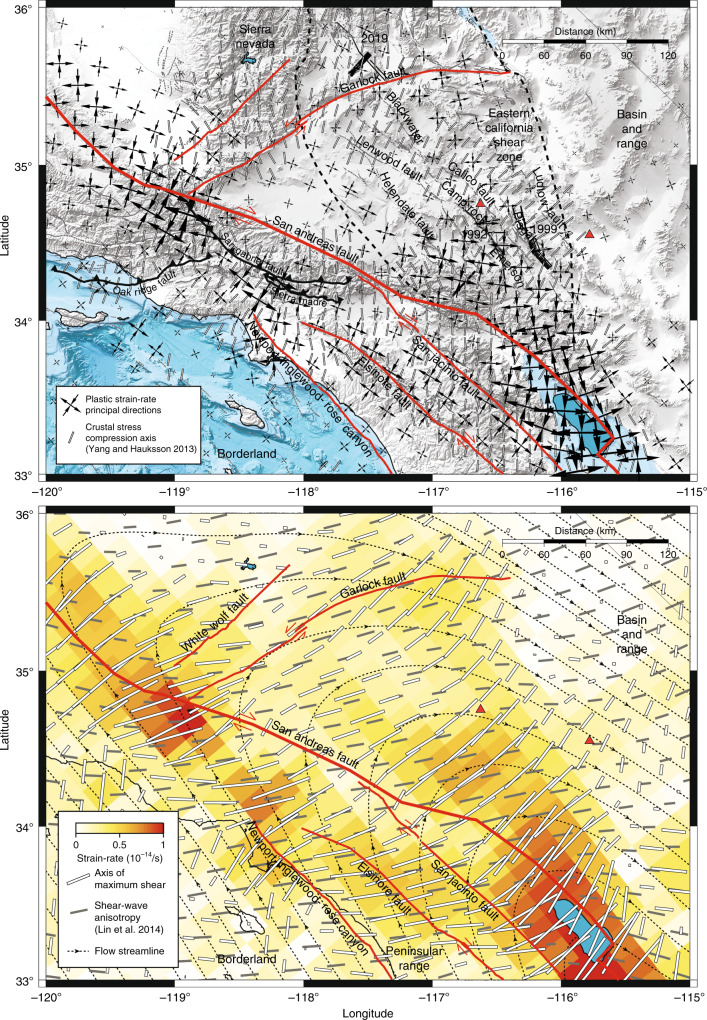
Principal strains, faults, seismicity, and seismic anisotropy. The top panel shows the principal strain direction of plastic strain-rate in relation to the orientation of strike-slip and thrust faults, and in relation with the compression axis of seismicity^67^. The bottom panel shows the direction of maximum shear of the plastic strain-rate and flow streamlines in relation to the orientation of seismic anisotropy^31^. Red lines indicate strike-slip faults. Black lines with chevrons indicate major thrust faults. Red triangles indicate quaternary volcanos. Grey lines indicate quaternary faults. The dashed contour indicates the approximative extent of the Eastern California Shear Zone.

The pattern of strain accumulation provides information about seismo-tectonic processes (Figs. 3 and 6), including the background stress, as the principal directions of strain-rate are identical to those of the driving deviatoric stress in the ductile region, and the direction of mantle flow at asthenosphere depths. In Mendocino county, the San Andreas fault is poorly oriented with the principal strain direction, contrarily to other faults inland. The strain principal directions are compatible with right-lateral strike-slip faulting on the San Andreas fault from Marin County, north of the Bay Area, to the triple junction at the Garlock fault and, further south, along the Coachella segment near the Salton Sea. Along the big bend of the San Andreas fault, the plastic strain-rate is smaller and less favorably oriented for strike-slip faulting, potentially compensated for by a weaker frictional resistance. The plastic strain-rate below the Garlock fault is optimally oriented for left-lateral strike-slip faulting from the junction with the San Andreas fault to Ridgecrest and Searles Valley, after which a restraining bend of the Garlock fault makes it less favorably oriented, compatible with the slip-rate of the Garlock decreasing to the east^65^. In the Mojave desert, plastic strain-rate is optimally oriented for right-lateral slip for all of the Helendale, Lenwood, Emerson, Camp Rock, Blackwater, Calico, Pisgaw, and Ludlow faults that together give rise to bookshelf faulting.

The background stress is also compatible with the source mechanisms of recent large Southern California earthquakes, including left-lateral and right-lateral slip for the 2019 Ridgecrest Mw 6.4 foreshock and Mw 7.1 mainshock, respectively^66^, left-lateral and right-lateral slip for the 1992 Big Bear and Landers earthquakes, respectively, and right-lateral slip for the 1999 Hector Mine earthquake. The compressive axis of crustal seismicity^67^ is perpendicular to major thrust faults in the Transverse Range and oblique to strike-slip faults in the Mojave block, but generally aligned with the compressive axis of plastic strain-rate. An even better alignment is found with the surface geodetic strain^67^, indicating a small rotation of the stress tensor towards the surface, presumably due to the short-range influence of faulting in the brittle layer. These results indicate that deep deformation dictates the large-scale stress patterns in the brittle layer that can be relaxed by different types of faults of various orientations^61^, each producing earthquakes with a specific focal mechanism.

## Discussion

Geodetic constraints on lower-crustal and mantle strain accumulation illuminate how shallow brittle crust and the deeper plastic layer accommodate regional deformation consistently with significant interaction. Plastic strain accumulation in northern California spreads from west to east across the San Andreas, Maacama, and Bartlett Springs fault systems in the Coastal Range and across the San Andreas, Hayward, and Calaveras fault systems in the Bay Area. In the central segment, the San Andreas fault sits above a similarly wide shear zone that stretches from the Oceanic and Rinconada fault zones to the west to the Great Valley thrust system to the east. In southern California, the deep shear zone is distributed over a wide region that spans across the Newport-Inglewood-Rose Canyon, Elsinore, and San Jacinto fault systems and the Coachella section of the San Andreas fault, possibly extending farther offshore. These results are compatible with the view that a fault network coalesces to a broad zone of distributed flow in the ductile layer^68^, with a separation distance for faults commensurate with the depth of the brittle layer. Theoretical considerations indicate that the thickness of parallel fault strands depends on the viscosity contrast between the lower and upper crusts^69^. However, the partitioning of fault slip to accommodate the underlying mantle flow can be accomplished by a set of parallel strike-slip faults, like in the northern and southern ends of the San Andreas fault system, or by a combination of strike-slip and en échelon folds and reverse faults, like in the central section. These results contrast with the simplistic view that major faults exist in isolation and extend from the surface to great sub-crustal depths with a similar degree of localization.

The broad deformation zone associated with a major restraining bend at asthenosphere depths impacts California tectonics from the Transverse Range to the Eastern California Shear Zone. A wide return flow surrounds the northern termination of the Salton trough shear zone, extending to north of the Garlock fault (Figs. 3 and 6b), where the flow direction rotates from SE-NW on the Pacific side, to W-E below the Mojave desert, and to NW-SE on the North American side. The flow pattern may shed light on the orientation of shear wave splitting in southern California. The development of seismic anisotropy in the upper mantle is thought to represent lattice preferred orientation of olivine minerals along the direction of maximum shear^70^, which can be estimated assuming a stationary flow by integrating the plastic strain-rate along the streamlines^71^. Considering a stable configuration of the San Andreas fault system in the last 4–6 Myr with particle velocity of 25 mm/yr and an average strain-rate of 5 × 10^−15^/s corresponds to 90–120 km of particle motion along the current streamlines building up about 95% plastic strain, enough to eliminate any previous texture. Computing synthetic seismic anisotropy is outside the scope of this work, but if the plastic strain-rate is sufficiently uniform upstream of the streamline, the direction of maximum shear of the current plastic strain-rate may represent a good proxy for the fast axis of shear wave splitting. From the California Borderland and Peninsular Range to the Mojave desert, where these conditions are met, there is good agreement with seismic observations^31^. However, shear wave splitting measurements have low depth resolution and may be sensitive to deformation below the asthenosphere, like a counter flow driven by the sinking of the Farallon slab^71^.

**Fig. 7:**
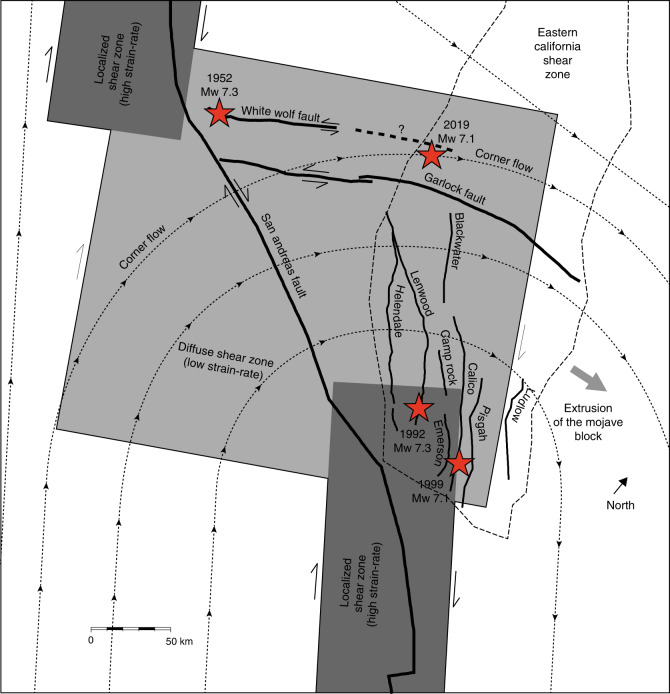
Evolution of fault activity due to extrusion of the Mojave block. Deformation in the Mojave is associated with corner flow distributed over a wide shear zone that accommodates the restraining step-over between more localized shear zones below the Carrizo and Coachella sections of the San Andreas fault. Faults that escaped out of the shear zone, like the Ludlow fault, become inactive. As the extrusion of the Mojave block continues, the White Wolf fault may materialize the triple junction at the big bend of the San Andreas fault. The stars represent large earthquakes in the last century.

The broad return flow below the most major step-over of the San Andreas fault also drives the eastward extrusion of the Mojave block. The advection of the eastern blocks of the San Andreas-Garlock unstable triple junction explains the formation of a new left-lateral shear zone along the White Wolf fault zone, which hosted the Mw = 7.3 Kern County earthquake in 1952^72^, and may eventually supplant the role of the Garlock fault for the regional block kinematics (Fig. 7). The extrusion of the Mojave block perpendicular to plate motion at a rate of  ~17 mm/yr is responsible for the peculiar long-term evolution of fault activity in the Mojave desert with increasing cumulative slip from west to east, as faults move past a stable sub-crustal shear zone^73^. The easternmost fault, the Ludlow fault, which simultaneously holds the largest cumulative offset and the smallest Holocene slip-rate, is no longer aligned with the Salton trough shear zone and is currently inactive. In contrast, the Calico and other faults to the west are currently still aligned with the Salton trough shear zone and have rapid slip-rates^42^. Even though a secondary zone of strain accumulation seems to connect the Eastern California Shear Zone and the Walker Lane with an average strain-rate of the order of 1 × 10^−15^/s, the major plate-boundary shear zones sit beneath the Salton trough and the Central section of the San Andreas fault, separated by a 120-km-wide restraining bend. The alignment of Mojave block faults with the deep Salton trough shear zone appears to be controlling their slip history.

**Fig. 8:**
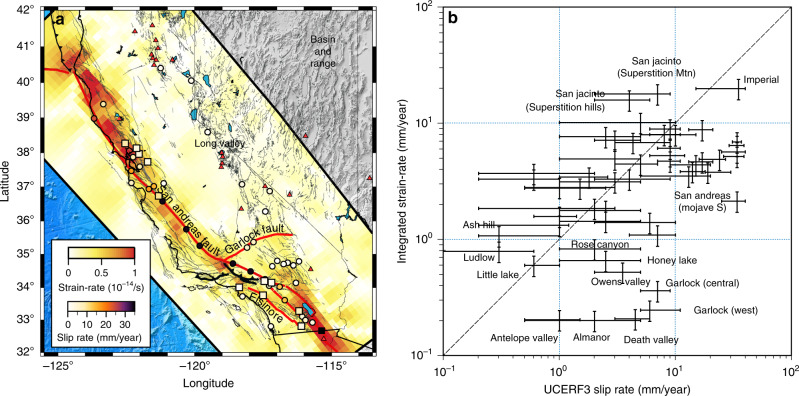
Anelastic strain accumulation and long-term slip-rate on California faults. **a** Spatial distribution of distributed strain and the location of paleo slip-rate on strike-slip fault segments cataloged in Appendix B of the Uniform California Earthquake Rupture Forecast (UCERF3; https://pubs.usgs.gov/of/2013/1165/). The squares indicate locations where the deep strain-rate immediately below the fault segment matches the long-term fault slip rate. Red triangles indicate quaternary volcanos. **b** Comparison of the UCERF3 slip rates with the integrated strain-rate below the corresponding fault segment.

The rate of seismicity on mature faults is controlled, among others, by the rate of stress accumulation, the size of the unstable-weakening region, and the style of faulting, all of which challenging to unravel. Plastic strain accumulation at lower-crustal and upper-mantle depths contributes to loading the seismogenic layer by elastic coupling. In addition, kinematic boundary conditions require the long-term relative displacement of blocks bounding the shear zone to be the same in the brittle and ductile layers. We investigate this relationship by comparing the long-term fault slip-rates on vertical or sub-vertical strike-slip faults collected in Appendix B of the Uniform California Earthquake Rupture Forecast^3^ with the offsets of the underlying volume elements in the fault-perpendicular direction. There is a broad agreement between geological slip rates and integrated plastic strain-rate (Fig. 8), even though mantle shear zones are diffuse, with no strict boundaries. Simple predictions based on underlying plastic strain-rate fall within the uncertainties of field observations for the Hayward, Calaveras, north San Andreas, south San Andreas, Newport-Inglewood, Elsinore, and San Jacinto faults. Large deviations occur for faults of the Eastern California Shear Zone, presumably due to off-fault deformation in the Mojave block^42^ and the variable rates of the Garlock fault in the late Pleistocene, early Holocene^65^. The overall agreement between shallow (geological) and deep deformation confirms the kinematic compatibility between the brittle and ductile layers, implying a strong mechanical coupling between upper-crust deformation and lower-crust and asthenosphere flow.

**Fig. 9:**
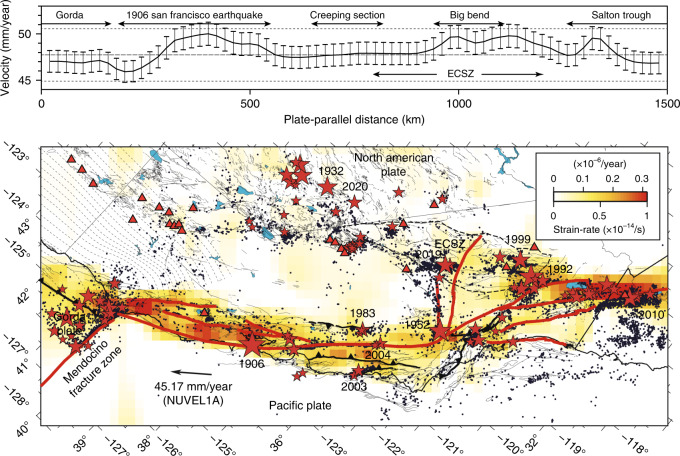
Strain partitioning across the San Andreas fault system. The top panel shows the plate parallel velocity from the integration of the shear component of the strain-rate tensor in the plate-perpendicular direction. The dashed lines indicate the average velocity and (1-*σ*) standard deviation of the Pacific plate relative to the North American plate from 18 different models, including REVEL-2000, NUVEL 1A, ITRF 2008, and MORVEL 2010 (refs. ^76–79^). The bottom panel shows the distribution of deep shear strain-rate in California from the Mendocino triple junction to the Salton trough, highlighting the strain partitioning primarily between the San Andreas fault and the Eastern California Shear Zone. The red stars and black circles show the hypocenter of historical earthquakes and earthquakes from moment magnitude 3 and above from 1990 to 2020 around California, respectively. The seismic catalog is obtained from the Northern California Earthquake Data Center (https://ncedc.org) and the Southern California Earthquake Data Center (https://scedc.caltech.edu). The gray dashed profiles correspond to the 5-km depth contours of the Cascadia megathrust. Red triangles indicate quaternary volcanos.

Assuming that plastic strain corresponds dominantly to simple shear, which holds true for most of California (Fig. 3), integration of the plastic strain in the plate-perpendicular direction shows a relative velocity between the Pacific and the North American plates in agreement with global reconstructions of relative plate motion, i.e., 49.7 ± 2.7 mm/yr (Fig. 9). Local deviations within the uncertainties occur along the plate boundary along the extent of the 1906 Mw = 7.9 San Francisco earthquake rupture, the Mojave block, and the Salton trough, which may be attributed to transient acceleration of mantle flow following large earthquakes, and in the Eastern California Shear Zone, where deformation is not in simple shear, involving a significant component of plate-perpendicular motion.

Plastic strain accumulation below the California margin is compatible with the relative motion of the North American and Pacific plates at geological time scales, with the long-term slip-rate of major faults of the San Andreas fault system, and with the contemporaneous surface deformation during the interseismic period. In addition, the trace of major faults, background seismicity, and major earthquakes are situated above regions of high plastic strain-rate (Fig. 9). These results indicate that deformation at the plate boundary is mechanically coupled from the crust down to the upper mantle, despite the possible weak strengths of the lower-crust and the asthenosphere. Mantle flow and crustal faulting form a cohesive geometric assembly where deep shear zones are overlaid by a kinematically consistent network of faults in the brittle layer. Kinematic compatibility is accomplished by slip partitioning in the brittle layer and strain partitioning in the ductile substrate, with geographic overlap.

As models of crustal dynamics can now represent the coupling between brittle and ductile processes^74,75^, imaging of deep deformation opens the door to more accurate models of crustal dynamics that assimilate a realistic distribution of mantle flow. Integration of brittle and ductile deformation processes into self-consistent mechanical representations will be key to better understand the geodynamics of the California margin.

## Methods

### Green’s functions

Any distribution of plastic strain in a viscoelastic medium causes deformation in its surrounding by elastic coupling. The details of the rheology in the ductile region is unspecified, but we assume that the flow is incompressible, driven by the deviatoric component of the stress tensor. With these assumptions, the plastic strain tensor ***ϵ*** can be associated the moment density **m** = 2*μ* ***ϵ***, where *μ* is the local shear modulus, and the equivalent body-force

1 $${\bf{f}}=-\nabla \cdot {\bf{m}}.$$

The resulting total displacement satisfies conservation of linear and angular momentum and can be obtained at any GPS point **x** of the domain by integration with the Green’s functions **G**(**x**; **y**), where **y** is the location of the forcing term, to automatically conform with the boundary conditions, following

2 $${\bf{u}}({\bf{x}})=\int_{\Omega }{\bf{G}}({\bf{x}};{\bf{y}})\cdot {\bf{f}}({\bf{y}})\ {\rm{d}}{\bf{y}}\,,$$

where Ω is the region that deforms plastically, potentially the whole domain. To image the distribution of plastic strain accumulation in California compatible with an inverse problem, we discretize the domain into semi-infinite cuboids that extrude downwards from a burial depth of *q*_3_  = 20 km. We approximate the total deformation field by superposition of the deformation caused by individual, non-overlapping volume elements of uniform plastic strain. Considering one volume element, the displacement field is given by

3 $${u}_{i}({x}_{1},{x}_{2},{x}_{3})=\int_{\partial \Omega }{G}_{ji}({x}_{1},{x}_{2},{x}_{3};{y}_{1},{y}_{2},{y}_{3})\ {m}_{jk}{n}_{k}\ {\rm{d}}{y}_{1}{\rm{d}}{y}_{2}{\rm{d}}{y}_{3},$$

where ∂Ω represents the boundary of the volume element, and repeated indices are summed, following Einstein’s convention. We use the Green’s function for an elastic half-space and the coordinate system is aligned with the orientation of the semi-infinite cuboid, depth positive down. We consider cuboids of length *L* and thickness *T* in the *x*_1_ and *x*_2_ directions, respectively, with the origin centered in the middle of a top edge. Expanding the integration over the four vertical surfaces and the top horizontal one, we can write

4 $${u}_{i}({x}_{1},{x}_{2},{x}_{3})= 	\int_{-T/2}^{T/2}\int_{{q}_{3}}^{\infty }{m}_{j1}{\left[{G}_{ji}({x}_{1},{x}_{2},{x}_{3};{y}_{1},{y}_{2},{y}_{3})\right]}_{{y}_{1} = 0}^{L}\ {\rm{d}}{y}_{2}{\rm{d}}{y}_{3}\\ \qquad\qquad\quad	+\int_{0}^{L}\int_{{q}_{3}}^{\infty }{m}_{j2}{\left[{G}_{ji}({x}_{1},{x}_{2},{x}_{3};{y}_{1},{y}_{2},{y}_{3})\right]}_{{y}_{2} = -T/2}^{T/2}\ {\rm{d}}{y}_{1}{\rm{d}}{y}_{3}\\ \qquad	-\int_{0}^{L}\int_{-T/2}^{T/2}{m}_{j3}\ {G}_{ji}({x}_{1},{x}_{2},{x}_{3};{y}_{1},{y}_{2},{q}_{3})\ {\rm{d}}{y}_{1}{\rm{d}}{y}_{2}.$$

We develop solutions for the displacement field using a numerical integration involving Gauss-Legendre quadrature along the *x*_1_ and *x*_2_ axes and double-exponential quadrature along the depth axis. For integration over depth, we use a change of variable that maps the semi-infinite interval into finite bounds. The displacement for semi-infinite cuboids of arbitrary strike angle from north at any coordinates in the reference system is obtained by translation and rotation of the displacement field. Surface displacement are associated with plastic strain; surface velocity with plastic strain-rate. Numerical codes that evaluate the displacement field are provided (see Code availability). The displacement fields for a buried volume element with non-zero plastic strain tensor components *e*_11_ and *e*_12_ are shown in Supplementary Fig. 2. Each volume element produces a similar displacement, accordingly shifted. Imaging the plastic strain accumulation can be thought of as a deconvolution of these displacement patterns. The diffusion of the surface velocity compared to the more localized extent of the plastic deformation represents the filtering effect of the elastic crust, which eventually limits the resolution of the inverse problem.

### Geodetic inversion method

We design the inversion of the surface velocity field by constructing the displacement kernels for each volume element of the mesh, complemented by other deforming surface and volume elements described in the main text. The matrices **L**_11_, **L**_12_, and **L**_22_ represent the three-component surface velocity vectors at the coordinates of the GPS stations due to the plastic strain tensor components ${\dot{{\bf{e}}}}_{11}$, ${\dot{{\bf{e}}}}_{12}$, and ${\dot{{\bf{e}}}}_{22}$, respectively, organized in a vector with one element per volume element in the mesh. For any distribution of horizontal plastic strain, the corresponding velocity field is given by the matrix-vector product ${\bf{L}}\ {({{\dot{{\bf{e}}}}_{11}}^{T},{{\dot{{\bf{e}}}}_{12}}^{T})}^{T}$, where the Green’s function matrix is defined as

5 $${\bf{L}}=\left({{\bf{L}}}_{11}-{{\bf{L}}}_{22}\qquad {{\bf{L}}}_{12}\right)\,,$$

enforcing an equal and opposite contribution of east-west and north-south uniaxial strain accumulation. The second-order deviatoric tensor (**e**_1_ ⊗ **e**_1_ − **e**_2_ ⊗ **e**_2_) is simply a 45^∘^ rotation of the tensor $({{\bf{e}}}_{1}\otimes {{\bf{e}}}_{2}+{{\bf{e}}}_{2}\otimes {{\bf{e}}}_{1})/\sqrt{2}$, so inverting for these two orientations simultaneously provides a basis to capture horizontal pure shear in any orientation. The velocity field contains a rigid-body motion representing rotation about an unknown pole. However, rotation about any arbitrary pole can be decomposed into a rotation about the origin of the coordinate system and a residual translation, both of which can be represented by a linear operator that can be inverted. We represent the rotation about the origin by the spin vector **w** = −*w***e**_3_ and the remaining translation by the vector **u**. The rigid-body component of the surface velocity is then given by the matrix-vector product ${\bf{R}}\ {(w,{u}_{1},{u}_{2})}^{T}$, with

6 $${\bf{R}}=\left(\begin{array}{ccc}-{{\bf{x}}}_{1}&{\bf{1}}&{\bf{0}}\\ +{{\bf{x}}}_{2}&{\bf{0}}&{\bf{1}}\end{array}\right).$$

Fault creep between the seismogenic zone and the brittle-ductile transition of major California faults is captured with 55 embedded surface elements, 5-km-wide and of varying lengths, with strike-slip motion. Near-surface creep along a central segment of the San Andreas fault is captured with 48 shallow embedded surface elements, 20-km-long and 5-km-wide, with strike-slip motion. Deep and shallow creep are related to the surface displacement by the matrices **F** and **C**, respectively. Additional volume elements with free plastic strain components ${\dot{{\bf{e}}}}_{11}$, ${\dot{{\bf{e}}}}_{12}$, ${\dot{{\bf{e}}}}_{22}$, and ${\dot{{\bf{e}}}}_{33}$ organized in a 7 × 7 × 3 element mesh extending 70 × 70 × 15 km around Long Valley caldera are used to model volcanic unrest and **M** is the matrix used to compute the associated displacements. The forward model is then given by **d** = **Gm**, where **d** is the vector of observations and the design matrix is given by ${\bf{G}}=\left({\bf{L}},{\bf{F}},{\bf{C}},{\bf{M}},{\bf{R}}\right)$. The inversion is stabilized with first-order and second-order Tikhonov regularization. A non-linear inversion procedure is used to enforce right-lateral shear in the plate-perpendicular direction and left-lateral shear in the plate-parallel direction. The residuals with the PBO crustal velocity field is shown in Fig. 4 and Supplementary Fig. 1.

## Supplementary information

Supplementary Information

## Data Availability

All data used in this study are publicly available. A reference to each dataset can be found in the figure captions and in the main text. The source data underlying Figs. 1, 3, 4, 5, 8a, and 9 and Supplementary Fig. 2a are provided as a Source Data file. Source data are provided with this paper.

## Source data

Source Data

## Code availability

The Matlab routines to compute Green’s functions for plastic strain in a semi-infinite cuboid in an elastic half-space are available at bitbucket.org/sbarbot/natcomm20. Source data are provided with this paper.
