# Supplementary material for: Mantle flow distribution beneath the California margin
Source: Nat Commun. 2020 Sep 8;11:4456. doi: 10.1038/s41467-020-18260-8 (PMC7479605; doi:10.1038/s41467-020-18260-8)
Supplement: Supplementary file 1 — Supplementary Information [file 41467_2020_18260_MOESM1_ESM.pdf]

Flow distribution beneath the California margin — Supplementary Information

Sylvain Barbot

Department of Earth Sciences, University of Southern California,
Los Angeles, CA 90089-0740, USA

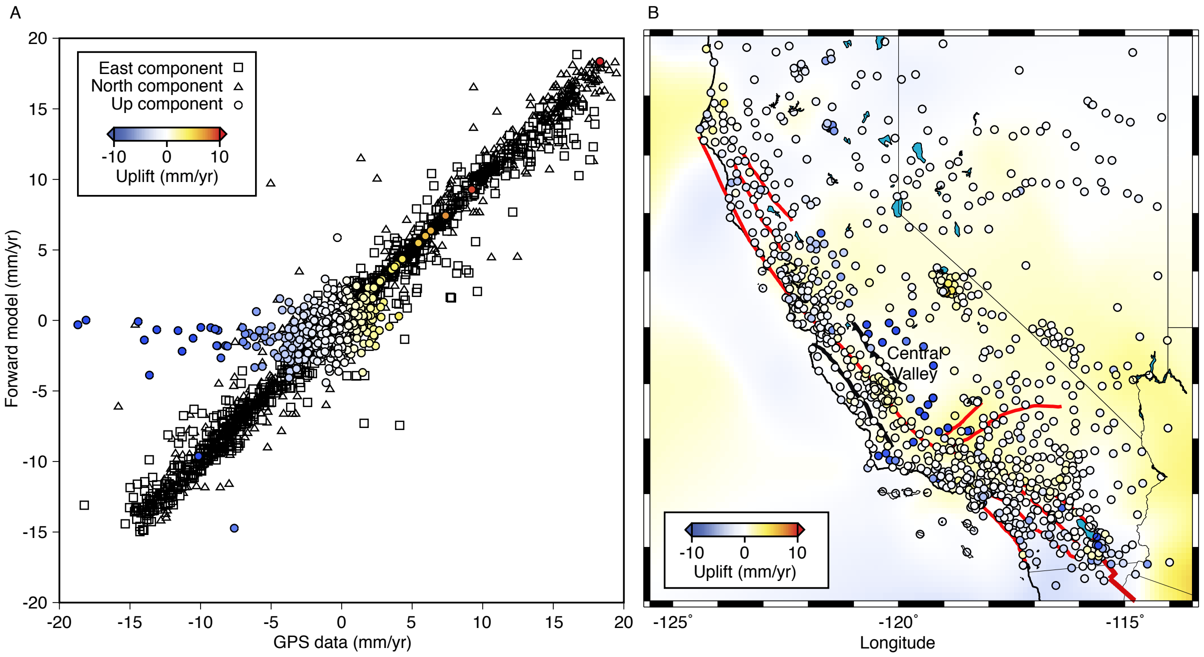

Supplementary Figure 1: Correlation between GPS data and model predictions. A) Observations and model predictions for the east (squares), north (triangles), and vertical (colored circles) components of the velocity field. B) Map view of vertical deformation for the data (colored circles) and forward model (background). Large subsidence in the Central Valley produce a few outliers.

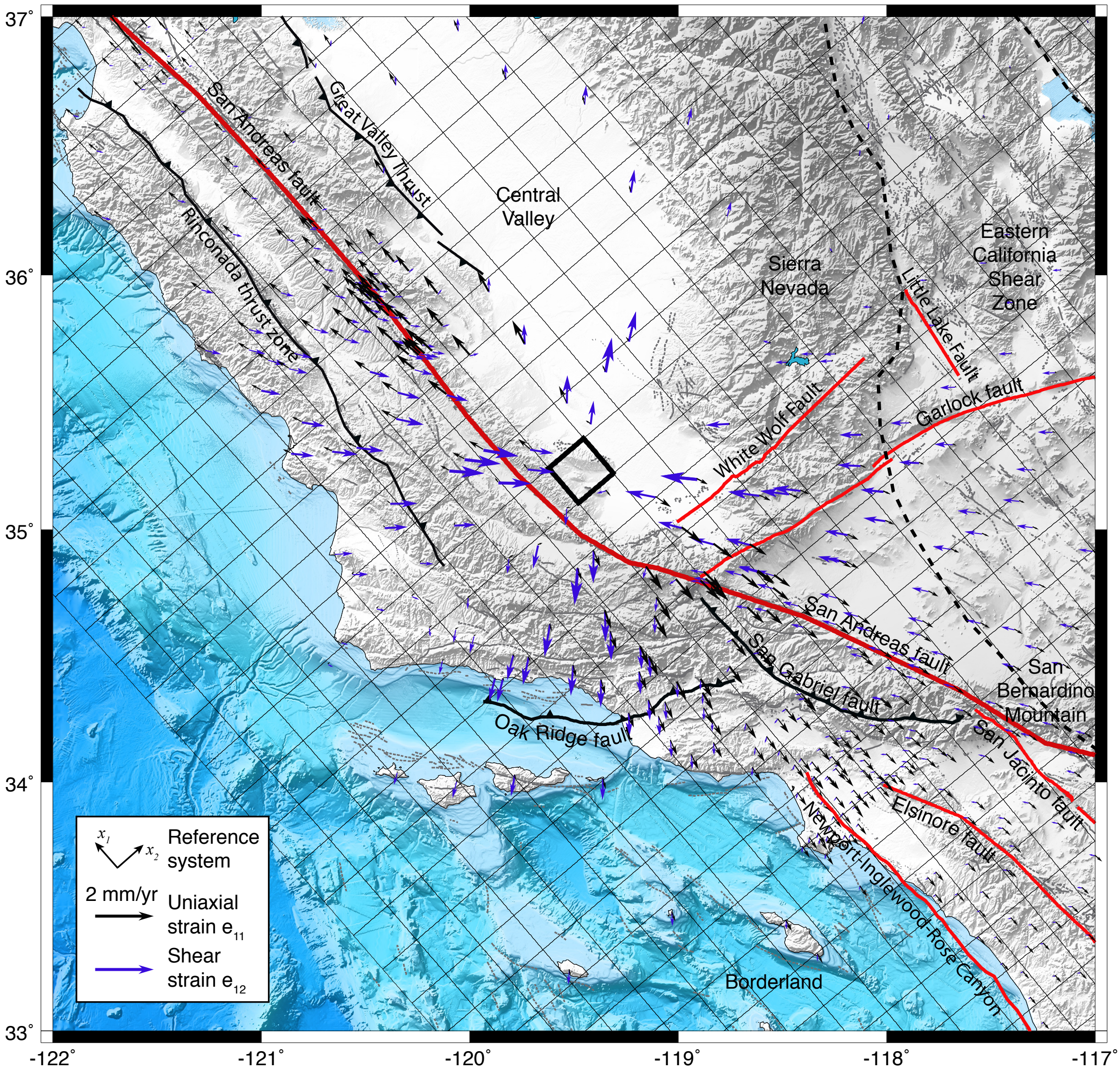

Supplementary Figure 2: Example Green's function. Surface deformation associated with distributed anelastic strain in a semi-infinite 20×20 km cuboid (black square) at 20 km depth. The black vectors correspond to the surface deformation for uniaxial horizontal strain along the approximately plate-parallel direction. The blue vectors are for the surface deformation due to a non-zero ϵ_{12} shear strain in the volume element. The underlying mesh of volume elements used to model distributed viscoelastic flow in and around California is shown in the background.
